# Mother and newborn survival according to point of entry and type of human resources in a maternal referral system in Kayes (Mali)

**DOI:** 10.1186/1742-4755-8-13

**Published:** 2011-05-10

**Authors:** Maman Dogba, Pierre Fournier, Alexandre Dumont, Maria-Victoria Zunzunegui, Caroline Tourigny, Safoura Berthe-Cisse

**Affiliations:** 1 Faculty of Medicine - Department of Public Health, University of Montreal, 1420 Mont-Royal Blvd., Montréal, Québec, H2V 4P3, Canada; 2 Research Center of the Centre hospitalier de l'Université de Montréal; CRCHUM, Canada; 3 Faculty of Medicine - Social and Preventive Medicine, University of Montreal, 1420 Mont-Royal Blvd., Montreal, Quebec H2V 4P3, Canada; 4 IRD; UR10 "Santé de la mère et de l'enfant en milieu tropical", Campus international UCAD/IRD, Route des Pères Maristes, BP 1386 Dakar-Hann-Sénégal; 5 Direction Régionale de la Santé, Kayes, Sénégal

**Keywords:** community health centers, referral system, emergency obstetric care, maternal mortality, stillbirth, probit, developing countries, Mali

## Abstract

**Background:**

Since 2001, a referral system has been operating in Kayes (Mali) to reduce maternal and perinatal deaths. Normal deliveries are managed in community health centers (CHC). Complicated cases are referred to a district health center (DHC) or the regional hospital (RH). Women with obstetric emergencies can directly access the DHC and the RH.

**Objective:**

To assess, in women presenting with an obstetric complication: 1) the effects of the point of entry into the referral system on joint mother-newborn survival; and 2) the effects of the configuration of healthcare team at the CHCs on joint mother-newborn survival.

**Method:**

Cross-sectional study of 7,214 women users of the referral system in the region of Kayes in 2006-2009. Bivariate probit equations were fitted to estimate joint mother-newborn survival. The marginal effects of the point of entry into the referral system and of the configuration of the healthcare team at the CHCs were evaluated with a probit bivariate regression.

**Results:**

Entering the referral system at the RH was associated with the best joint mother-newborn survival; the most qualified the CHCs team was, the best was mother-newborn survival. Distance traveled interacts with the point of entry and the configuration of the CHCs team. For women coming from far (over 50 km), going directly to the RH increased the probability of joint mother-newborn survival by 11.90% (p < 0.001) as compared with entry at the CHC. Entry at the CHC while coming from a distance of less than 5 km increased the likelihood of joint survival by 8.50% (p < 0.001). Among women who go first to a CHC, physician presence increased joint mother-newborn survival, compared with having no physician and fewer than three professionals. The size of the healthcare team at the CHC is significantly associated with mother-newborn survival only when distance traveled is 5 km or less.

**Conclusion:**

Mother-newborn survival in the Kayes maternal referral system is influenced by combined effects of the point of care, the skill configuration of CHC personnel and distance traveled.

## Background

More than 20 years after the launch of the Safe Motherhood Initiative and despite the recognition, in the Millennium Development Goals (MDG), of the importance of reducing maternal and perinatal mortality, progress has been very slow [1-3]. In the most affected countries, as in Mali (West Africa), to improve significantly mother and child health requires scaling up professional skilled care at childbirth for all women and improving the care environment for quick access to emergency obstetric care for complicated cases [4-7].

Due to its high maternal mortality ratio - 464 deaths per 100,000 live births [8] - Mali has made reducing maternal mortality a key health policy objective. One of its strategies is the evacuation and referral system (ERS). It is also aimed at preventing perinatal deaths, especially stillbirths, for which estimates are scarcely available in developing countries [9,10].

The referral system has been monitored and evaluated by the Kayes Regional Health Department in collaboration with the Mali National Health Department and the University of Montreal since 2004.

This study is part of that larger evaluation programme. It assesses the impacts on maternal mortality and stillbirths of the point of entry and the type of human resources in the referral system. It addresses two research questions: 1) what point of entry into the system is associated with the best rates of joint survival of mother and newborn; and 2) what is the role of the community health centers' (CHC) teams in the joint survival of mother and newborn?

## Setting

Mali, one the poorest nation in sub-Saharan Africa, has eight administrative regions including Kayes. The Kayes region has made a great effort to have a functional ERS in its seven health districts^a ^in accordance with national directives. Table 1 displays the demographic characteristics and the numbers of expected deliveries, in the seven districts of the Kayes region.

**Table 1:** General characteristics and selected health indicators of the districts of the Kayes region (2009)

	Bafoulabé	Diéma	Kayes	Kéniéba	Kita	Nioro	Yélimané	Total
Population^c^	209,383	180,284	423,140	194,416	385,769	208,882	159,214	**1,761,008**
Number of CHCs	39	21	46	22	45	27	23	**223**
District area (km^2^)	20,120	12,360	22,190	16,800	35,250	11,060	5,700	**123,849**
Expected deliveries^d^	10,469	9,014	21,157	9,721	19,288	10,444	7,961	**88,054**

^c ^From 1998 census data, calculated with the rate of natural increase.

^d ^Calculated as 5% of population.

The ERS has three components: a) upgrading of obstetric services; b) funding by community health fund; and c) improving transport and communication by ambulance and radio communication. The care component of the ERS is focused on *basic *emergency obstetric care (EmOC) and neonatal care in the CHCs (community health centers: first-level care) and *complete *EmOC and neonatal resuscitation in the DHCs (district health centers: second-level care) and in the RH (regional hospital: third-level care).

The CHCs handle normal deliveries. They vary considerably in the levels of training and numbers of human resources responsible for *basic *EmOC. Some CHCs have physicians, midwives and obstetric nurses; they may have up to eight persons. Other CHCs only have three persons: a nurse, a matron, and a caregiver. Due to shortages of human resources and the difficulty of attracting and retaining midwives at the peripheral level in Kaye's region, only eleven of the 223 CHCs had midwives in 2009. Rather, maternal care is provided mainly by matrons, who are chosen usually from the local community and trained minimally and locally. Matrons are not classified among skilled birth attendants, who should be

"*Trained to proficiency in the skills needed to manage normal (uncomplicated) pregnancies, childbirth and the immediate postnatal period, and in the identification, management and referral of complications in women and newborns"(*[11]* page 1)*.

In the DHCs of the six rural districts, there are general practitioners with surgical skills, such as for caesareans, as well as nurse anaesthetists, midwives and obstetric nurses. The DHC of Kayes' capital region only plays an administrative role in coordinating the transfer of women from CHCs to the RH. At the top of the regional healthcare pyramid, the RH, located in the capital region district, has obstetricians, physician anaesthetists, midwives and a more advanced technological platform.

**Figure 1:**
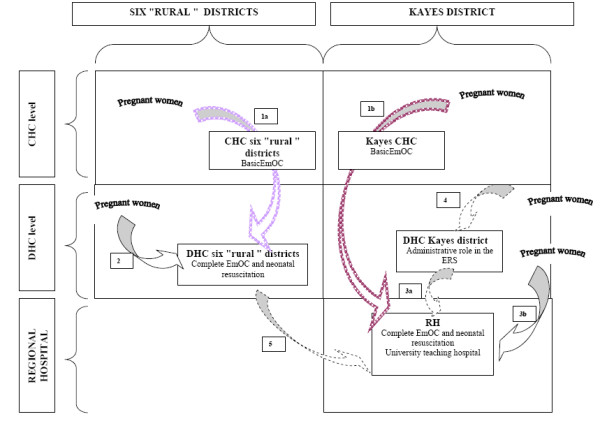
**Different points of entry in the Kayes Evacuation and referral System**.

Pregnant women are expected to enter the system at the CHC level for normal deliveries. If a complication arises that endangers the mother or the newborn, the woman is either referred to the DHC, in the six rural districts, or to the RH if she is in the Kayes district. Surgical management of obstetric emergencies and neonatal resuscitation are only available at the DHCs and the RH. These are respectively the first and the topmost levels of referral in the healthcare pyramid, although they can be used as local facilities by patients living nearby. Figure 1 outlines the various points of entry into the Kayes ERS.

## Method

### Study population

The study population was selected according to the following inclusion criteria: pregnant women residing in Kayes region, transferred from a CHC or directly admitted at the higher referral level (DHC or RH) between January 1, 2006, and December 31, 2009 for an obstetric complication confirmed by the referral level staff (Figure 1, paths 1a, 1b, 2, 3a and 3b) and who gave birth to a newborn weighting more than 500 grams during the current episode of care. In case of multiple pregnancies, only the first baby was included in the study.

Excluded from the study were women who entered the health system after home birth because the information on the newborn is not systematically collected in these cases and we could not assess the effects of the point of entry into the ERS on joint mother-newborn survival. Women who resided in border areas and countries, such as Koulikoro region, Senegal or Mauritania^b^, or had been transferred from a DHC to the RH (Figure 1, path 5) or gave birth at the Kayes district DHC (Figure 1, path 4) were also excluded because they did not benefit from the ERS.

The second research question, which was the role of the primary care teams on the joint mother-newborn survival, was addressed with the subgroup of women transferred from a CHC to DHC (Figure 1 paths 1a and 1b).

Data sources:

a) An ongoing system of registering all obstetric emergencies has been implemented since 2004 (GESYRE: *Gestion du Système de Référence Évacuation */Management of the Evacuation Referral System). Information is systematically recorded on patients who use the ERS: age, location of residence, medical and surgical history, diagnosis of complications, treatments provided and outcome of care for mothers and babies. In each DHC and at the RH, there were specially trained staffs responsible for gathering data in the GESYRE. The research team supervised directly the data collection twice a year and updated the database by double checking and classifying the various cases into direct and indirect obstetric complications based on the ICD-10 [12]. When no diagnosis was available, women were classified based on risk factors and treatment provided. Women who had received a caesarean or a forceps delivery were coded as presenting an obstetric complication, as were those whose risk factors suggested a direct or indirect obstetric emergency such as a uterine rupture. Cases without diagnosis who were registered with risk factors such as "age 16 years old and less" were not labelled obstetric complication if they had normal deliveries, as were those whose diagnosis was "normal labour and delivery" and who did not correspond to the above-mentioned criteria.

b) Regular surveys allowed us to collect quarterly data on the human, administrative and financial resources of the CHCs, the DHCs and the RH. Any change in the care team was noticed in order to assign women to the correct types of care team present at the CHCs where they were admitted.

Training sessions with Kayes EmOC staff were held to harmonize the definitions of obstetric emergencies. Major obstetric complications such as dystocia (prolonged labour, uterine rupture), haemorrhage, hypertensive diseases of pregnancy, infections, and all other complications requiring a caesarean or a hysterectomy were labelled obstetric emergencies [13].

### Statistical analysis

The outcomes examined by the study are mother survival and newborn survival at birth. Mother and newborn survival are dichotomous variable. Deaths included are those recorded at the referral level before the hospital discharge (institutional death).

The main explanatory variable of interest for the first research question was the point of entry into the ERS (women who enter the ERS at the CHCs level - CHClevel -, women who go directly to the DHC -DHClevel- and women who go directly to the RH -RHlevel-). For the second research question relating only to women who enter the ERS at the CHC level, the main explanatory variable was the configuration of CHC care team based on staff numbers and qualifications (CHC that has a physician - CHC MD; CHC that has no physician and three or fewer care providers - CHC noMD ≤ 3P; CHC that has no physician and with more than three care providers - CHC noMD > 3P) and the traveled distance between the CHCs and the higher referral level (DHC or RH).

For both research questions, distance was an important identified explanatory variable due to the emergency context of care and also because it could approximate accessibility to care. In this study, distance travelled was approximated by the distance between the CHC and the DHC or RH and does not take into account the distance from the women residence to a care center (5 km or less; from 6 to 50 km; more than 50 km).

Control variables were selected from well-known risk factors for maternal death and stillbirth. Their possible confounding role in the association between, on one hand maternal death, stillbirth and the point of entry; and, on the other hand, maternal death, stillbirth and the configuration of CHC care team is reported in the literature [14-16]. The control variables were diagnosis (uterine rupture or prolonged labour; haemorrhage; eclampsia or pre-eclampsia; other direct and indirect causes), age (women age 16 years and under; those 17 to 34 years old, and those age 35 years and more), previous caesarean section (yes or no), blood transfusion during the current episode of care (yes or no), and caesarean delivery for current pregnancy (yes or no).

A place variable, "District", was created to differentiate between patients who were treated in Kayes from others.

Uni and bivariate analysis were carried out, using chi2 tests to analyse the differences in sample distribution by outcomes. As we were interested in considering simultaneously the association of survival of the mother and the newborn at birth, a bivariate probit analysis was undertaken. A bivariate probit analysis is sued for modelling two binary dependent variables jointly as a function of some explanatory variables and controlling factors [17]. The joint probability of mother and newborn survival was first modeled with the simple marginal effects of the covariates. Second, interaction terms between explanatory variables were included in the two bivariate probit analyses.

Descriptive analyses were performed using SPSS version 15 (SPSS Inc., Chicago IL) and the probit models were adjusted using Stata version 9. A level of significance of 0.05 was used for all the analyses.

## Results

The first research question estimates the effects of the point of entry into the ERS on the joint survival of the mother and the newborn at birth. To answer this question, 7,214 women for whom data were complete for all variables in the model were retained for the analyses. The 464 women for whom information was missing on one of the variables differ from those with complete data, in terms of the proportion who go to CHCs (53.7% vs. 36.4%, p < 0.001). No significant differences were found for the other variables.

Table 2 shows the sample's characteristics and the distribution of the joint probability of mother-newborn survival by all covariates for the 7,214 women.

**Table 2:** Distribution of the mother and child survival by point of entry and relevant variables (N = 7,214)

	N (%)	Mother and newborn joint survival (%)	p-value^e^
Total (N = 7,214)		5494 (76.2)	
**Point of entry into the ERS**			
*CHClevel*	3 873 (53.69)	70.64	< 0.001
*DHClevel*	1 519 (21.06)	78.20	
*RHlevel*	1 822 (25.26)	86.17	
**Distance**			< 0.001
*5 km or less*	3 162 (43.83)	82.51	
*From 6 to 50 km*	2 188 (30.33)	72,03	
*More than 50 km*	1 864 (25.26)	70.22	
**Diagnosis**			< 0.001
*Uterine rupture and dystocic/prolonged labor*	3 612 (50.07)	83.14	
*Hemorrhage*	898 (12.45)	47.99	
*Eclampsia/Pre-eclampsia*	615 (8.53)	72.68	
*Other*	2 089 (28.96)	77.21	
**Treatment: current caesarean**			< 0.001
*No*	2 889 (40.05)	68.60	
*Yes*	4 325 (59.95)	81.20	
**Treatment: transfusion**			< 0.001
*No*	6 823 (94.58)	78.36	
*Yes*	391 (5.42)	37.59	
**Previous caesarean section**			< 0.001
*No*	6 605 (91.56)	74.58	
*Yes*	609 (8.44)	93.27	
**Age**			< 0.001
*16 years or less*	966 (13.39)	81.26	
*35 years and more*	980 (13.58)	66.73	
*17 to 34 years*	5 268 (73.02)	76.97	
**District**			< 0.001
*Rural districts*	4 256 (59.00)	73.00	
*Kayes*	2 958 (41.00)	80.70	

^e ^Chi-square test; level of significance 0.05

A total of 7,042 mothers and 5,553 newborns were alive (respectively 96.6% and 77%). The effects of the explanatory variables on the mother and newborn survival separately are shown in Table 3. The overall Wald statistic test significantly confirmed the good performance of the bivariate probit model (Wald Chi Square = 1143, p = 0.0000; data not shown), evaluating the adjusted effect of level of entry and/or the traveled distance on the joint probability of mother-newborn survival. The test of the term errors showed that the two outcome variables were positively and significantly correlated and then the probability of mother survival was associated with the newborn survival (rho = 0.45, Chi Square = 96.47, p = 0.0000). Changes in point of entry and/or traveled distance were significant in explaining the joint mother-newborn joint survival (data not shown).

**Table 3:** Coefficients for the effects of explanatory variables on the mother and newborn survival (N = 7,214)

Variables	Mother survival	Newborn survival
**Point of entry into the ERS**		
*CHClevel*	Ref	Ref
*DHClevel*	0.06	0.18**
*RHlevel*	- 0.12	0.30**
**Distance**		
*More than 50 km*	Ref	Ref
*5 km or less*	0.37**	0.27**
*From 6 to 50 km*	0.16*	0.09*
**Diagnosis**		
*Uterine rupture and dystocic/prolonged labor*	Ref	Ref
*Hemorrhage*	-0.20*	-0.85**
*Éclampsia/Pre-eclampsia*	-0.84**	-0.24**
*Other*	-0.01	-0.23**
**Treatment: current caesarean**		
*No*	Ref	Ref
*Yes*	-0.32**	0.41**
**Treatment: transfusion**		
*No*	Ref	Ref
*Yes*	-0.61**	-0.77**
**Previous caesarean section**		
*No*	Ref	Ref
*Yes*	0.37	0.54**
**Age**		
*35 years and more*	Ref	Ref
*16 years or less*	0.32*	0.41**
*17 to 34 years*	0.18*	0.22**
**District**		
*Rural districts*	Ref	Ref
*Kayes*	0.38**	0.15**

* p < 0.05; ** p < 0.01

Once integrating the interaction terms, there was a significant combined effect of traveled distance and the point of entry on the mother-newborn survival. Table 4 shows the adjusted effects of the interaction terms between distance and point of entry on the mother-newborn survival. When compared to women who come from more than 50 km enter the ERS at the CHC' level, the joint mother-newborn survival was higher in women who directly go to the DHCs or the RH. Among women who entered at the CHC level, the prognosis was improved as distance decreased (p < 0.001). Among women who came from far (more than 50 km), only those who went to the RH had a better joint mother-newborn survival (11.90% higher than those going to CHCs p < 0.001). Finally, going to the RH from a distance of 5 km or less was associated with the best probability of joint survival (14.51% higher than the reference group, p < 0.001).

**Table 4:** Effects (in percentage) of the interaction term between distance and point of entry on the joint mother and newborn survival (N = 7,214)

	Point of entry	Coefficients	95% CI of coefficients	p
> 50 km	CHClevel	Ref	---	----
	DHClevel	3.66	[-1.83 - 9.15]	0.191
	RHlevel	11.90	[5.99 - 17.81]	0.000
6-50 km	CHClevel	3.12	[0.47 - 5.77]	0.021
	DHClevel	7.96	[3.53 - 12.39]	0.000
	RHlevel	10.71	[4.99 - 16.43]	0.000
0-5 km	CHClevel	8.50	[8.36 - 11.64]	0.000
	DHClevel	13.34	[10.05 - 16.63]	0.000
	RHlevel	14.51	[11.03 - 17.99]	0.000

^++^adjusted for age, distance, diagnosis, transfusion, current caesarean, prior caesarean and district

The effects of the configuration of care team in the CHCs from which the women were referred were analyzed in the second research question. For this purpose, 3,869 women with complete data on all selected variables were included in the analysis. Of the 173 women for whom information was missing, a greater proportion was admitted in Kayes than among those whose data were complete (52.0% vs. 29.3%; p < 0.001). For the other variables, both categories of women were comparable.

The total number of alive mothers were 3,753 (97%); and 2,768 (71.5%) newborn were alive. The proportion of mother and newborn who simultaneously survive were 67.83%, 76.02% and 73.72% among CHC noMD ≤ 3P, CHC noMD > 3P and CHC MD respectively (p < 0.001 on chi-square test).

The overall Wald statistic test significantly confirmed the good performance of the bivariate probit model (Wald Chi Square = 1543, p = 0.0000) when testing the adjusted effects of the configuration of CHCs care team and/or the traveled distance to the health care center on the joint probability mother-newborn survival. The test of the term errors showed that the two outcome variables were positively and significantly correlated and then the probability of mother survival was associated with the newborn survival (rho = 0.47, Chi Square = 63.86, p = 0.0000).

Interaction terms between the distance traveled and the configuration of CHCs care teams were significant. Table 5 reports on the adjusted effects of the interaction term between distance and configuration of CHCs care teams on the joint mother-newborn survival.

**Table 5:** Effects (in percentage) of the interaction term between configuration of CHC care team and distance on the mother and newborn survival (N = 3,869)

	Type of care team	Coefficients	95% CI of coefficients	p
> 50 km	CHC noMD ≤3P	Ref	---	----
	CHC noMD > 3P	0.38	[-6.99 - 7.75]	0.919
	CHC MD	6.07	[0.74 - 11.38]	0.025
	CHC noMD ≤3P	3.29	[-2.50 - 6.83]	0.069
6-50 km	CHC noMD > 3P	4.31	[-2.94 - 11.57]	0.244
	CHC MD	9.34	[2.52 - 16.18]	0.007
0-5 km	CHC noMD ≤3P	4.76	[-1.88 - 11.41]	0.160
	CHC noMD > 3P	14.87	[10.13 - 19.61]	0.000
	CHC MD	9.44	[2.71 - 16.16]	0.006

^++^adjusted for age, distance, diagnosis, transfusion, current caesarean, prior caesarean and district.

A combined effect of distance and configuration of care team was observed, but the effects of distance became less important in a CHC MD. When compared to women who travelled for more then 50 km and want to CHC noMD ≤3P, the join mother-newborn survival was better in those who go to CHC MD whatever the distance traveled. The highest care team effect was found in CHC noMD > 3P when distance traveled was 5 km or less (14.87% higher than that of the reference group; p < 0.001). But the size of care team at CHC is associated with mother-newborn survival only when distance traveled is 5 km or less.

## Discussion

This study shows a combined effect of distance and point of entry into the ERS on the probability of joint mother-newborn survival when age, diagnosis, transfusion, and prior and current caesarean are adjusted for. The RH level achieved better outcomes of care for the mother and the newborn than did the DHC level. Moreover, receiving care at the RH appears more beneficial, even for women who come from far, than going first to the CHC; this is the case even when the distance between the CHC and the final level of care is 5 km or less.

The better outcome of care associated with the RH is to be expected, since an enabling environment with skilled birth attendants and permanent EmOC is crucial for life-saving interventions in obstetric emergencies [4]. Thus, women going to the RH have a better prognosis than those going to DHCs. These two levels of care differ in staff configuration and care environment. The RH has not only a more advanced technological platform, but also obstetricians, midwives and other healthcare personnel in greater numbers and with more training than are found in DHCs. This structural difference leads to a different organization of services, with available on-call coverage and full-time obstetric services in the RH [18,19]. For those who first go to the CHC and develop obstetric complications, life-saving interventions are not available at the CHCs, and they need to be transferred to a better equipped center. This might not be a straightforward process because the attending staff must decide on the transfer. Delays in decision-making could also contribute for the less good outcomes that occur even when CHCs are near the DHC or the RH. Such delays are not explored in this study. Nevertheless, the types of care teams present at the CHC also influence the joint mother-newborn survival.

Indeed, this study confirms that size of the health care team can influence maternal and perinatal outcomes. When the care team is made up of more than three professionals, the probability of joint mother-newborn survival is increased, but only when the traveled distance is short (5 km or less). As recommended in the literature, maternal intrapartum care provided by a team of professionals, preferably midwives, improves health outcomes [20]. Such teams can simultaneously look after many women and share the workload, thus improving the quality of care. However, as in Kayes, they can also organize the administrative aspects of referral for transfer to a higher level of care. Nevertheless, the protective effect of the staff number effect is limited to a short distance.

This study shows also that physician presence at the CHC is associated with a significantly better mother-newborn outcome, whatever the distance, and the effect of the distance is less important in CHCs with a physician.

The protective effect of physicians' presence at the CHC level is not surprising in Kayes' specific context of care, as they are, at the peripheral level, the highest skilled personnel. Women who go to a CHC where there is no MD are treated by nurses and matrons whose skills in maternal care are often less than those required from a skilled birth attendant. The use of less skilled personnel, due to staff shortages, may be a temporary alternative for normal deliveries. Nevertheless, more skilled personnel are required for detecting and managing efficiently obstetrical complications. The protective effects of physician' presence may be related either to better technical quality of care or to interrelational consequences of the physician's presence.

In terms of quality of care, it may be that physicians make early referrals for women who present with a pregnancy at high risk for maternal or infant death, or they may detect complicated cases early and provide suitable obstetric and neonatal care. They might also manage more adequately women coming from far and who are in worse clinical conditions. In fact, having more skilled staff has long been associated with better outcomes in obstetric complications. Historical studies cite the professionalization of midwives as one of the major determinants in reducing maternal mortality in developed countries [21,22]. Conversely, the failure of the traditional birth attendants' policy is partly due to lack of training and to the increased monitoring required to support their performance [21,23]. A comparative analysis of maternal morbidity and mortality in two districts of Senegal confirmed these conclusions by showing that maternal morbidity was better diagnosed by midwives and physicians than by nurses and matrons [13]. Doctors may have more theoretical knowledge [24], but midwives demonstrated better practical skills at managing normal deliveries. In every case, beyond the staff's qualifications, it is primarily their skill in managing complications that matters; yet in Kayes, most pregnancies are managed by matrons.

The interrelational consequences of a physician's presence at the CHC can be observed at all three levels of care. First, the referral might be better accepted when it is made by a more qualified professional. Bossyns et al. [25] analyzed the referral system as a social process that requires the understanding and support of both patients and personnel. Their analysis showed that lack of acknowledgement of the referral's added value or feelings of powerlessness among health professionals were major obstacles to a good referral system. Second, the presence of a physician, by improving a CHC's reputation, can result in early use of maternal services [19]. Finally, besides having an impact on the patient, the presence of a physician can change the relationships among the different hierarchical levels and produce a differential reactivity among the personnel. Thus, patients referred or evacuated by a physician would be treated sooner than others. This explanatory hypothesis should be explored further.

Even though the RH is associated with the best mother-newborn outcomes, implementing a hospital in every local community is neither a cost-effective nor feasible public health option due to financial constraints and shortages of qualified human resources [20,26-28]. Thus, a functional referral system is theoretically designed to improve access to quality EmOC and therefore enhance mother-child survival. A previous study in Kayes showed that the benefits of using the referral system were greater when all the components, including transport, are used [29]. The present study extends the understanding of important determinants of a referral system's efficacy to the quality of the care team and thus suggests ways of improving the Kayes maternal system. As midwives are hardly found in the CHCs of Kayes, measures to upgrade human resources skills at the first level and to train, attract and retain midwives in rural areas must be implemented without delay [4-6,30,31].

This study evaluated the effects of the point of entry and the CHC care team configuration in the ERS on the probability of joint mother-newborn survival. Taking into account, at the same time, the women's individual characteristics allowed us to control for some important confounding factors of maternal mortality and stillbirths. Certain limitations must nevertheless be noted. First, we did not assess the role of traveled time and of the time between the onset of delivery and seeking care. Yet distance traveled, as a proxy of accessibility, remains an important factor in mother-newborn survival. Second, this study did not explore other individual factors such as the woman's education and her power to make decisions. However, the inclusion of a place variable might have captured some important rural-urban differences. Finally, the inclusion in the analysis of data collected over four years supports the study's conclusions. Third, survival estimates in this study are probably an over estimation of the true survival rates because women who have come to the hospital after giving birth at home have been excluded and also because in hospital mortality is an underestimation of the mortality rate; as derived from other studies in hospital mortality is about 80% of the whole maternal mortality.

## Conclusion

Although the CHCs do not have the technological platform that would allow them to provide specialized emergency obstetric care, the presence of qualified personnel at this peripheral level is a determining factor for mother-child survival, especially for women who live in more remote areas and access to the health system in a poorest health condition. To really reduce maternal and perinatal mortality, major efforts must be directed toward these women living in rural and remote areas. It may be neither cost-effective nor advisable to have a physician in every CHC but upgrading the skills of the CHC personnel may be an effective short-term strategy. Placing less qualified staff in health facilities should be done cautiously, even in a functional referral system. These results should inspire further, more in-depth studies to identify and understand the mechanisms that produce the effects observed. Then it will be possible to replicate good practices in healthcare teams and to find the staff configurations that perform best within each country's specific context.

## Competing interests

The authors declare that they have no competing interests.

## Authors' contributions

MD and PF conceived the study and carried out its design, data analysis, as well as data interpretation. CT and SBC carried out the data gathering and analysis. MVZ, AD and PF participated in substantial statistical and editorial revisions of this paper. All authors read and approved the final manuscript.

## Note

^a^The district in which the regional capital is located and six other districts in somewhat more rural areas. In this paper, we refer to these as "the six rural districts".

^b^The solidarity funds do not include these border regions in their funding schemes for the Kayes ERSs.
